# Biological Activities of *Sargassum* Algae Mediated ZnO and Co Doped ZnO Nanoparticles as Enhanced Antioxidant and Anti-Diabetic Agents

**DOI:** 10.3390/molecules28093692

**Published:** 2023-04-25

**Authors:** Hassan Ahmed Rudayni, Abdelrahman M. Rabie, Malak Aladwani, Lina M. Alneghery, Gasem M. Abu-Taweel, Wail Al Zoubi, Ahmed A. Allam, Mostafa R. Abukhadra, Stefano Bellucci

**Affiliations:** 1 Department of Biology, College of Science, Imam Muhammad bin Saud Islamic University, Riyadh 11623, Saudi Arabia; 2 Petrochemicals Department, Egyptian Petroleum Research Institute, Nasr City, Cairo 11727, Egypt; 3 Department of Biology, College of Science, Jazan University, P.O. Box 2079, Jazan 45142, Saudi Arabia; 4 Materials Electrochemistry Laboratory, School of Materials Science and Engineering, Yeungnam University, Gyeongsan 38541, Republic of Korea; 5 Zoology Department, Faculty of Science, Beni-Suef University, Beni-Suef 65214, Egypt; 6 Materials Technologies and Their Applications Lab, Geology Department, Faculty of Science, Beni-Suef University, Beni-Suef 65214, Egypt; 7 Geology Department, Faculty of Science, Beni-Suef University, Beni-Suef 65214, Egypt; 8 INFN, Laboratori Nazionali di Frascati, E. Fermi 54, 00044 Frascati, Italy

**Keywords:** seaweed, ZnO, cobalt doping, antioxidant, antidiabetes

## Abstract

Brown macroalgae (BMG) were used as carriers for ZnO (ZnO/BMG) and cobalt-doped ZnO (Co-ZnO/BMG) via facile microwave-assisted hydrothermal synthesis. The multifunctional structures of synthesized composites were evaluated as enhanced antioxidant and anti-diabetic agents based on the synergistic effects of ZnO, Co-ZnO, and BMG. BMG substrate incorporation and cobalt doping notably enhanced the bioactivity of the synthesized ZnO nanoparticles. As an antioxidant, the Co-ZnO/BMG composite exhibited highly effective scavenging properties for the common free reactive oxygen radicals (DPPH [89.6 ± 1.5%], nitric oxide [90.2 ± 1.3%], ABTS [87.7 ± 1.8%], and O_2_^●−^ [46.7 ± 1.9%]) as compared to ascorbic acid. Additionally, its anti-diabetic activity was enhanced significantly and strongly inhibited essential oxidative enzymes (porcine α-amylase (90.6 ± 1.5%), crude α-amylase (84.3 ± 1.8%), pancreatic α-glucosidase (95.7 ± 1.4%), crude intestinal α-glucosidase (93.4 ± 1.8%), and amyloglucosidase (96.2 ± 1.4%)). Co-ZnO/BMG inhibitory activity was higher than that of miglitol, and in some cases, higher than or close to that of acarbose. Therefore, the synthetic Co-ZnO/BMG composite can be used as a commercial anti-diabetic and antioxidant agent, considering the cost and adverse side effects of current drugs. The results also demonstrate the impact of cobalt doping and BMG integration on the biological activity of ZnO.

## 1. Introduction

The recent unexpected increase in the number of patients with diabetes worldwide represents a critical health issue, as an increase to 366 million patients by 2030 is predicted. Diabetes is currently the seventh leading cause of death [1,2,3]. This clinical pancreatic syndrome is classified into two main types: (A) diabetes mellitus type 1 (T1-DM) and (B) diabetes mellitus type 2 (T2-DM). The latter is widely distributed and is likely to comprise 90% of diabetes cases by 2030 [1,4]. T2-DM is a serious metabolic disorder that results in a significant generation of reactive oxygen species (ROS) or oxidizing radicals at abnormal concentrations and an increase in blood glucose levels (post-prandial hyperglycemia) [5,6]. Post-prandial hyperglycemia is the underlying reason for several clinical complications such as kidney failure, cardiomyopathy, coronary, heart disease, polyphagia, morbidity, mortality, polydipsia, glycosuria, nephropathy, retinopathy, and neuropathy [7,8,9]. However, the generation of ROS causes significant pathophysiological effects, such as oxidative stress, and a considerable decline in antioxidant functions, which, in turn, have adverse effects on lipid peroxidation and insulin resistance, in addition to their damaging effect on blood vessels and cellular organelles [6,8,10,11].

Biguanides, miglitol, sulfonylureas, acarbose, thiazolidinedione, and voglibose are commonly used effective commercial anti-diabetic drugs that exert regulatory effects on ROS levels and post-prandial hyperglycemia [6,8,12]. Unfortunately, several adverse side effects (diarrhea, abdominal distention, hepatotoxicity, severe hypoglycemia, and meteorism) have been reported for most of the above-mentioned drugs, in addition to their high costs [6,13]. Therefore, several types of innovative multifunctional structures of several bioactive chemical groups have recently been developed as enhanced anti-diabetic and antioxidant agents that can be applied effectively as scavengers and inhibitors of oxidative enzymes [6,14]. Among the assessed synthetic materials, bioactive metal oxides, such as ZnO, CuO, and NiO, exhibit significant antioxidant and anti-diabetic effects. These effects have been attributed to their unique physicochemical properties (surface reactivity and surface area), low manufacturing cost, remarkable biological activity, and significant biocompatibility, as well as their considerable therapeutic and theranostic potentials [14,15,16].

Among these metal oxides, synthetic ZnO nanoparticles and their composites have been extensively applied as promising bioactive materials that exhibit significant biocompatibility, nontoxicity, and antioxidant and anti-diabetic properties [17,18,19]. Zinc is a vital bioactive element in the human body, especially during the production of nucleic acids and proteins [15,19]. Additionally, synthetic zinc oxides and their base materials are extensively used in pharmaceutical, medical, and biological industries as antioxidant, anticancer, hypoglycemic, and antibacterial agents, in addition to their vital role as drug delivery systems and use in tissue engineering industries [15,20]. Recent literature has widely documented the valuable antioxidant properties of ZnO nanoparticles, which can effectively regulate mitochondrial respiration, inhibit oxidant enzymes, and reduce oxidizing reactive oxygen free radicals [6,14].

The accumulation affinities of synthetic ZnO nanoparticles via van der Waals forces, as well as their reported superficial properties and rapid electron-hole recombination rates, adversely affect the generation efficiency of free radicals and, in turn, their photocatalytic and biological activities [14,21]. Several studies confirmed a significant influence of the synthesis technique, production conditions, crystallite size, and morphology of ZnO on its biomedical and biological activities [15,18,22]. Surface functionalization and hybridization of synthetic ZnO using different chemical and physical methods have been reported to be highly effective techniques for enhancing its bioactivity, biocompatibility, antibacterial activity, genotoxicity, and antioxidant properties [15,18,23]. The commonly used modification techniques involve (1) doping synthetic ZnO with other transition metals, (2) loading ZnO into an effective carrier or substrate, (3) incorporating synthetic ZnO into biocompatible biopolymers in composites, and (4) synthesizing chemical complexes of ZnO and bioactive phytochemicals [14,19,24,25].

Previous studies have shown the positive impact of organic, inorganic, and organic/inorganic substrates, as well as common macro- and micro-marine algae, especially brown macroalgae (*Sargassum* species), on the physicochemical and catalytic properties of ZnO [21,26]. Seaweed (brown macroalgae) is a promising renewable bioresource with easy cultivation, high production rates, economic viability, and therapeutic and nutritional value [27,28,29]. Brown macroalgae are extensively used as sources of several vital biologically active compounds that exhibit significant antibiotic, antifungal, antibacterial, anticancer, anti-inflammatory, and antioxidant properties [29,30,31]. These compounds include terpenoids, polyphenols, polysaccharides, sargaquinoic acids, plastoquinones, sargachromenols, steroids, and glycerides [32].

Therefore, the application of brown macroalgal microstructures as a substrate for pure or modified ZnO is expected to result in innovative multifunctional composites with significantly enhanced biomedical and biological applications. To the best of our knowledge, the biological activity and biomedical applications of synthetic ZnO nanoparticles under microwave irradiation, as well as the elemental doping of ZnO with brown macroalgae (*Sargassum* species), have not yet been fully investigated. Therefore, this study is the first to investigate the biological activities of pure ZnO and Co-doped ZnO (Co-ZnO) nanoparticles and their composites with brown macroalgae as potential antioxidant and anti-diabetic agents. We investigated the scavenging efficiencies of the synergetic forms of the synthetic structures for common oxidative radicals and their inhibitory effects on essential enzymes.

## 2. Results and Discussion

### 2.1. Characterization of the Synthetic Structures

**Figure 1:**
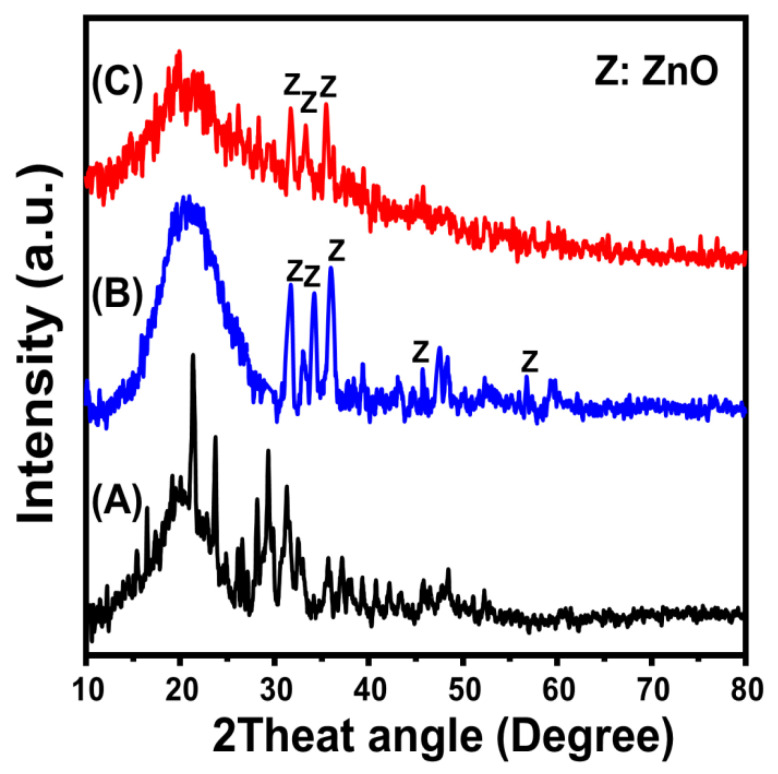
XRD patterns of brown macro-algae substrate (BMG) (A), synthetic ZnO/BMG composite (B), and synthetic Co-ZnO/BMG composite (C).

The obtained XRD patterns of the brown macroalgae incorporated as a substrate in addition to the ZnO/BMG and Co-ZnO/BMG composites are presented in Figure 1. The observed pattern of the algae exhibits the broad peak typical of amorphous organic materials (2theta angle of approximately 22°) in addition to several peaks possibly related to impurities and salts (Figure 1A). The ZnO/BMG pattern clearly demonstrated several identification peaks of crystalline ZnO (hexagonal wurtzite) at 31.68° (100), 34.51° (002), 36.27° (101), 47.8° (102), and 56.57° (110) (JCPDS no. 65-3411; JCPDS no. 36-1451) (Figure 1B). The considerable deviation in the reported position of the ZnO identification peak compared to the JCPDS cards, in addition to the remarkable detection of the broad peak of the algal substrate, confirmed successful integration of the supported ZnO and the organic formwork of the algae. The observed pattern of Co-ZnO/BMG demonstrated a notable reduction in the intensities of the difficult-to-detect peaks of ZnO in the pattern of ZnO/BMG, in addition to a considerable deviation of these peaks to lower 2theta angles (Figure 1C). Such structural observations, in addition to the lack of detection of clear peaks related to the formation of cobalt oxide, indicate the existence of cobalt as a doping element within the structure of ZnO rather than as a separated oxide phase [33]. The average crystallite size of the synthesized ZnO phase in ZnO/BMG was 51.7 nm, whereas that of Co-ZnO/BMG was 44.3 nm. Such a reduction in the crystallite size reflects the impact of the doped Co element, as it can hinder the progressive growth of ZnO crystals by forming Co-O-Zn surface chemical groups, in addition to significantly inhibiting the agglomeration potential of the synthetic crystals [34]. 

**Figure 2:**
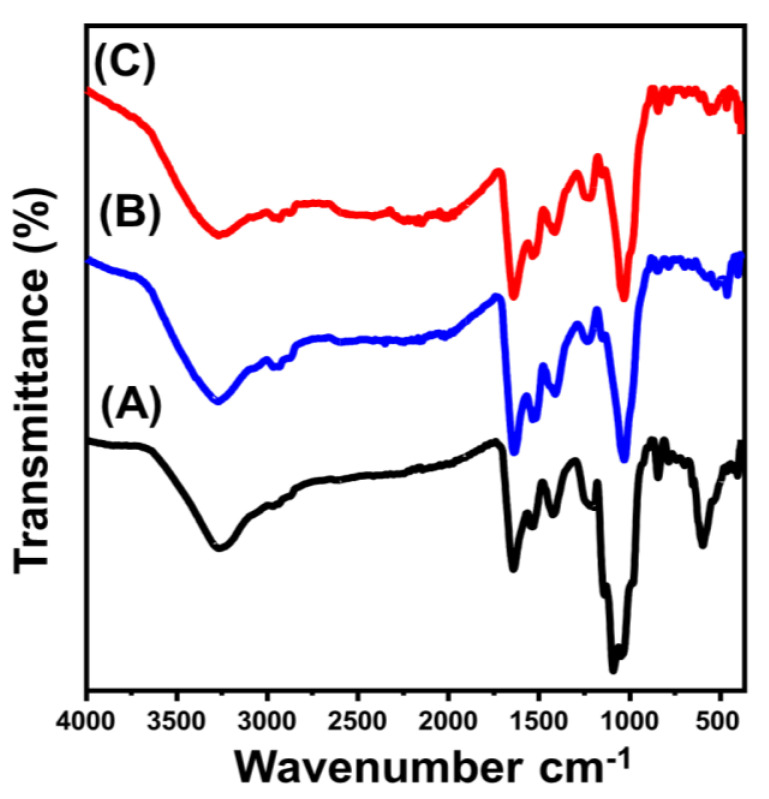
FT-IR spectra of brown macro-algae substrate (BMG) (A), synthetic ZnO/BMG composite (B), and synthetic Co–ZnO/BMG composite (C).

Previous findings were also based on the FT-IR spectra of the synthetic materials compared to the obtained spectrum of brown macroalgae (Figure 2). The detected broad band with an area from 3200 to 3600 cm^−1^ implies the stretching vibration of N–H and O–H bonds within the alginate, lipid, protein, and cellulose compounds of the algae structure (Figure 2A) [35,36]. Additionally, other organic chemical groups were clearly identified from the spectrum of BMG, such as aliphatic –CH_2_– (2920 cm^−1^), asymmetrical carboxylate groups (1643 cm^−1^), symmetrical carboxylate groups (1447 cm^−1^), sulfate esters within the fucoidan structure (1256 cm^−1^), sulfonic acids (SO_3_) (1161 cm^−1^), alcoholic groups (1064 and 1021 cm^−1^), and mannuronic groups of alginate structure (863 cm^−1^) (Figure 2A) [36,37,38]. The recognized spectra of ZnO/BMG and Co-ZnO/BMG demonstrated slight deviations in the positions of the previously identified bands of the algae. Moreover, new reduced bands at approximately 460 cm^−1^ were detected, implying O–Zn–O stretching (Figure 2B,C) [21,39]. This finding indicates a clear interaction or formation of complexes between the chemical groups of the algal chemical structure and the supported ZnO nanoparticles [21,40].

**Figure 3:**
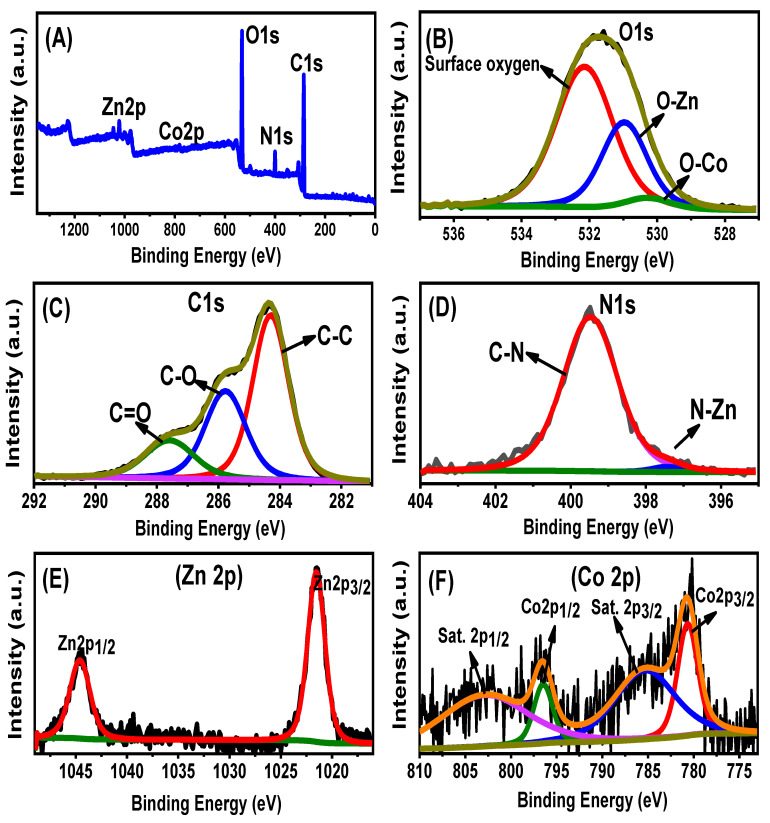
XPS spectra of the synthetic Co-ZnO/BMG composite (**A**) including the O1s spectra (**B**), C1s spectra (**C**), N1s spectra (**D**), Zn spectra (**E**), and Co spectra (**F**).

The chemical structures identified based on the reported chemical groups significantly matched the elemental compositions according to the EDX spectra. The EDX spectrum of ZnO/BMG confirmed the presence of Zn (24.56%), O (40.9%), and ZnO and C mixture (34.54%) as the main components of the algae. The EDX spectrum of Co-ZnO/BMG confirmed the presence of Zn (22.16%), O (39.4%), and Co (3.2%) in the composition of Co-doped ZnO and C (35.24%) as the main composition of the algae. The XPS spectrum of the synthesized Co-ZnO/BMG particles was investigated to confirm the doping process, integration reactions, and oxidation states (Figure 3). The full scan spectrum confirmed the elemental composition of the composite, which contained N, C, Co, Zn, and O (Figure 3A). The three clear peaks in the O1s spectra indicate surface oxygen (532.12 eV), O-Zn (530.96 eV), and O-Co (530.27 eV) (Figure 3B) [26]. The recognized three spectral peaks of C1s identify the bonds of C=O (287.58 eV), C-O (285.78 eV), and C-C (284.3 eV) (Figure 3C) [41]. The two clear peaks observed in the N1s spectra imply the bonds of C-N (399.47 eV) and N-Zn (397.39 eV) (Figure 3D). The spectra of zinc show the presence of Zn with two binding energies at 1021.5 eV (Zn 2p_3/2_) and 1044.6 eV (Zn 2p_1/2_) (Figure 3E) [42]. Moreover, the clear peaks at 780.6 eV (785.3 eV satellite peak) and 796.46 eV (802.67 eV satellites peak) identify the state of the doped cobalt as Co 2p_3/2_ and Co 2p_1/2_, respectively (Figure 3F) [43].

**Figure 4:**
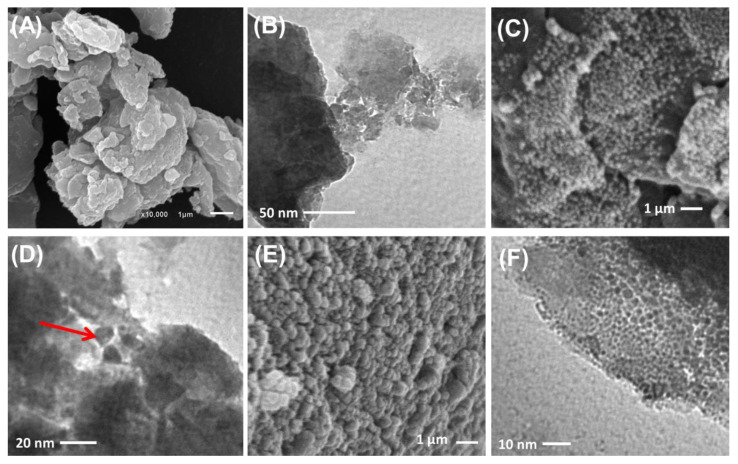
SEM image of brown macro-algae substrate (**A**), HRTEM image of brown macro-algae substrate (**B**), SEM image of synthetic ZnO/BMG composite (**C**), HRTEM image of synthetic ZnO/BMG composite (**D**), SEM image of synthetic Co-ZnO/BMG composite (**E**), and HRTEM image of synthetic Co-ZnO/BMG composite (**F**).

SEM and HRTEM analyses reflected the successful decoration of algae with synthetic ZnO and Co-ZnO nanoparticles (Figure 4). The algal particles used as substrates appeared as massive compacted particles without definite outlines, which are common for natural particles in both the SEM (Figure 4A) and HRTEM (Figure 4B) images. Regarding the synthetic ZnO/BMG and Co-ZnO/BMG structures, the surface of the massive algal particles in the SEM images appeared to be highly decorated or ornamented with nanoparticles of semi-spherical forms related to the loaded ZnO (Figure 4C,D) and Co-ZnO grains (Figure 4E,F). The HRTEM images also confirmed the SEM observations and indicated the significant impedance of the synthetic ZnO (Figure 4D) and Co-ZnO grains (Figure 4F) as nanoparticles within the matrix of the incorporated particles of brown macroalgae as a substrate. The determined surface areas of BMG, ZnO/BMG, and Co-ZnO/BMG particles were 5.4, 11.3, and 10.4 m^2^/g, respectively.

### 2.2. Antioxidant Properties

#### 2.2.1. Nitric Oxide Scavenging

**Figure 5:**
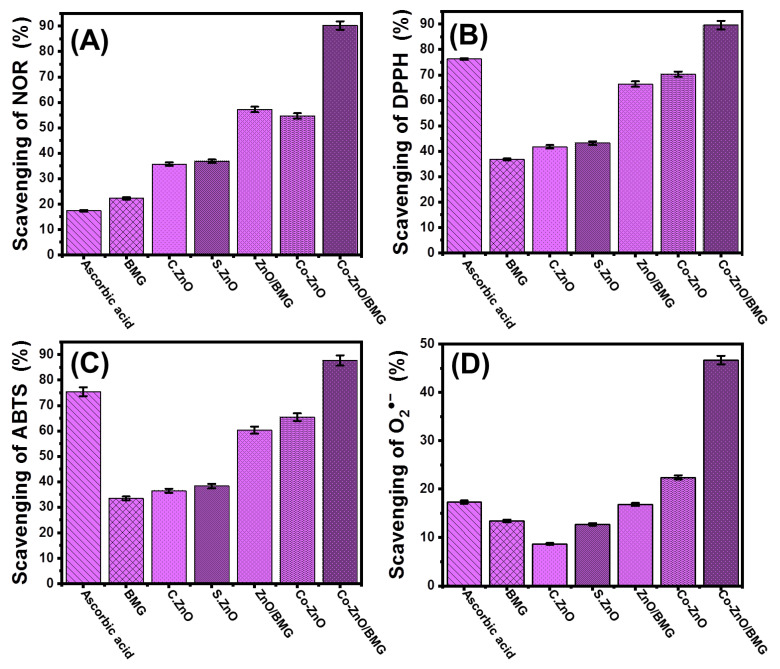
The antioxidant activities of BMG, commercial ZnO (C.Zn), synthetic ZnO (S.Zno), Co-ZnO, ZnO/BMG, and Co-ZnO/BMG structures; (**A**) nitric oxide scavenging activities; (**B**) DPPH scavenging activities; (**C**) ABTS scavenging activities; and (**D**) superoxide radical scavenging activities.

The extensive generation of reactive oxygen, especially during aerobic respiration and electron transportation, causes notable oxidative stress and numerous degenerative diseases [44]. Among these reactive species, the gaseous free nitric oxide radical (NOR) and its irregular generation may be associated with DNA fragmentation, cell cytotoxicity, neuronal cell death, and cell damage [6,45,46]. Metal oxides commonly used as antioxidant agents have demonstrated significant NOR scavenging properties. The scavenging activity of the synthetic ZnO nanoparticle against NOR was notably enhanced after cobalt doping and after the application of BMG particles as a substrate (Figure 5A). The estimated scavenging percentage of BMG, commercial ZnO (C.ZnO), free synthetic ZnO (S.ZnO), and Co-ZnO for NIR were 22.2 ± 1.3%, 35.7 ± 1.5%, 36.8 ± 1.4%, and 54.7 ± 1.7%, respectively (Figure 5A). Therefore, the cobalt doping process increased the scavenging activity of S. ZnO by approximately 17.9%, which is 19% higher than that of C. ZnO and matches the previously reported results in the literature (Figure 5A) [25,47]. This was attributed to the reported surface electrons of the Co-ZnO nanoparticles, which effectively pair with the lone pairs of hydroxyl radicals [26,47]. Electron-supplying capacity is an essential controlling factor that significantly affects the antioxidant properties of metal oxides [48]. Therefore, the incorporation of Co into the synthetic ZnO structure increased the generation efficiency of surface free electrons, which improved the free radical scavenging performance.

The incorporation of the BMG substrate forming ZnO/BMG and Co-ZnO/BMG composites resulted in remarkable enhancement in the NOR scavenging activity to 57.2 ± 1.4% and 90.2 ± 1.3%, respectively (Figure 5A). The reported activities of ZnO/BMG and Co-ZnO/BMG were significantly higher than those of free ZnO and Co-ZnO, respectively. The detected enhancement effect of the BMG substrate was attributed to its positive impact on the exposure and interactive interfaces of the loaded ZnO nanoparticles, in addition to its reduction effect on the agglomeration affinities between the supported particles [21]. Moreover, the chemical compounds of brown macroalgae exhibit notable antioxidant activity, which increases the interaction rates with free active oxidizing species. Therefore, the synthetic Co-ZnO/BMG composite can be used as a more effective antioxidant against NOR compared to the separated phases of ZnO, Co-ZnO, and BMG as well as the ascorbic acid control (21.6 ± 1.3%) (Figure 5A).

#### 2.2.2. DPPH Radical Scavenging

DPPH scavenging by ZnO/BMG and Co-ZnO/BMG was tracked in synergetic tests and compared to that of BMG, C.ZnO, S.ZnO, and Co-ZnO. These marked behaviors matched the properties observed during the scavenging reactions of NOR (Figure 5B). The DPPH scavenging percentage determined using Co-ZnO/BMG was 89.6 ± 1.5% which was clearly higher than the recognized efficiency using ZnO/BMG (66.4 ± 1.8%) by approximately 23.2% (Figure 5B). Based on the recognized activities by BMG (36.7 ± 1.66%), C.ZnO (41.7 ± 1.6%), S.ZnO (43.2 ± 1.2%), and Co-ZnO (70.3 ± 1.1%), the cobalt doping modification in combination with the incorporation of the BMG substrate strongly enhanced the scavenging activity of ZnO for the DPPH radical which also is higher than the activity of ascorbic acid standard (76.3 ± 1.3%) (Figure 5B). The scavenging of the DPPH radical by the surfaces of synthetic metal oxides occurs via electron (e^−^)/proton (H^+^) transfer reactions towards the organic framework structure of the DPPH radical [49,50]. The doped cobalt ions within the ZnO crystals induce the generation rate of surface electrons and charge separation efficiency, which enhances the activity of Co-ZnO as compared to ZnO. In addition, the negatively charged groups of the BMG chemical compounds might accelerate the charge separation reactions and enhance the effect of the algal substrate on the interactive interfaces between the DPPH radical and bioactive ZnO structures [51].

#### 2.2.3. ABTS Radical Scavenging

ABTS scavenging tests have been used as an effective indicator of the antioxidant potential of synthetic materials, especially composites or hybrid structures, based on the actual decline in the concentration of the diffused ABTS cation radical (ABTS^●+^). Synthetic structures exhibiting effective hydrogen-donating antioxidant mechanisms can be used as scavengers of ABTS^●+^ radicals. Therefore, the synthetic metal oxides either as pure forms or modified products by doping reactions or hybridization by the suitable substrate are highly endorsed as scavengers for ABTS^●+^. The detected ABTS^●+^ scavenging percentage of BMG (33.4 ± 1.8%), C.ZnO (36.4 ± 1.4%), S.ZnO (38.3 ± 1.3%), Co-ZnO (65.4 ± 1.8%), and ZnO/BMG (60.3 ± 1.77%) was lower than the value detected for the ascorbic acid control (75.4 ± 1.1%) (Figure 5C). Impeding the Co-doped ZnO nanoparticles into the matrix of the BMG (Co-ZnO/BMG) resulted in a significant enhancement in its scavenging activity for ABTS^●+^ radical to 87.7 ± 1.8%, i.e., higher than the recognized activity of ascorbic acid (Figure 5C).

#### 2.2.4. Superoxide Radical Scavenging

The superoxide anion (O_2_^●−^) is a reactive oxygen radical generated within the cellular organelles such as mitochondria transforms immediately into other oxidizing species such as hydroxyl radicals (^●^OH) and H_2_O_2_. The uncontrolled and non-regulated generation of O_2_^●−^ and its transformed products cause severe pathophysiological conditions in addition to numerous hazardous health risks and degenerative diseases, including the cellular destruction of DNA, RNA, and protein [52,53]. Generally, the biological system of the human body has natural defenses against the diffused O_2_^●−^ as well as its transformed products and their oxidative stresses to maintain safe physiological homeostasis. However, various diseases have adverse effects on the biological responses in human organs during the generation of defense functions, depending on the type or the quantities required to control radical levels. Therefore, the effective scavenging of O_2_^●−^ by the innovative and biocompatible multifunctional structures has been strongly recommended. The determined BMG scavenging percentage for O_2_^●−^ (13.4 ± 1.8%), C.ZnO (8.7 ± 1.2%), S.ZnO (12.7 ± 1.5%), and ZnO/BMG (16.8 ± 1.3%) were lower than the reported scavenging efficiency of the ascorbic acid control (17.3 ± 1.3%) (Figure 5D). However, the cobalt-doped ZnO (Co-ZnO) (22.4 ± 1.4%) and its integrated products with BMG (Co-ZnO/BMG) (46.7 ± 1.9%) showed significantly higher activity than the commercially used ascorbic acid (Figure 5D).

### 2.3. Anti-Diabetic Properties

#### 2.3.1. Porcine Pancreatic α-Amylase Inhibition Assay

**Figure 6:**
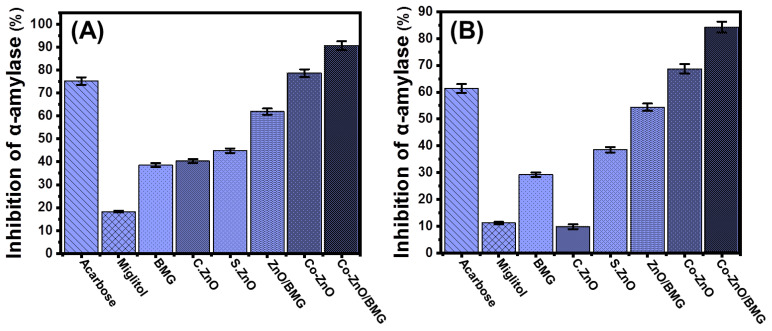
The α-amylase inhibition activities of BMG, commercial ZnO (C.ZnO), synthetic ZnO (S.ZnO), Co-ZnO, ZnO/BMG, and Co-ZnO/BMG structures; (**A**) porcine pancreatic α-amylase enzyme; and (**B**) murine pancreatic α-amylase enzyme.

The anti-diabetic activities of BMG, S.ZnO, Co-ZnO, ZnO/BMG, and Co-ZnO/BMG were assessed in synergetic studies to determine their inhibitory effects on α-amylase. The α-amylase enzyme is an essential and dominant digestive enzyme that causes significant breakdown of long chains of carbohydrate polymers, such as starch, into simple forms, such as maltose, which also immediately transforms into glucose species [6]. Therefore, the synthetic structures that display significant and rapid inhibition against the α-amylase enzyme will cause a considerable decline in the breakdown rates of the complex carbohydrates and, in turn, slow the absorption of dietary starches and limit spikes in blood sugar levels [54]. Therefore, post-prandial hyperglycemia in diabetes can be safely and significantly controlled [24]. The α-amylase enzyme inhibition percentage by Co-ZnO (78.5 ± 1.7%) and Co-ZnO/BMG (90.6 ± 1.5%) demonstrates remarkable anti-diabetic activity as compared to the commercial drugs used as standards (acarbose (75.2 ± 1.7%) and miglitol (18.3 ± 1.4%)) (Figure 6A). In addition, the prepared S.ZnO (44.8 ± 1.5%) and ZnO/BMG structures (61.9 ± 1.8%) exhibited enhanced inhibition effects on the α-amylase enzyme as compared to BMG (38.6 ± 1.8%), C.ZnO (40.3 ± 1.6%), and miglitol (18.3 ± 1.4%) (Figure 6A). The reported enhancement in the anti-diabetic activity of the prepared ZnO was attributed to cobalt-doping functionalization and the incorporated BMG substrate. The BMG substrate effectively reduced the agglomeration potential of synthetic ZnO and Co-ZnO on its surface, which enhanced the exposure and interactive interfaces of their particles with enzymes [14,47,54,55]. The agglomeration and aggregation of metal oxides adversely affect their biological activities and inhibit oxidative enzymes [47]. Based on the inhibitory activity of Co-ZnO and Co-ZnO/BMG on the porcine pancreatic α-amylase enzyme, they can be recommended as enhanced, low-cost, effective, and safe anti-diabetic agents compared with expensive commercial drugs that commonly lead to side effects [56,57].

#### 2.3.2. Murine Pancreatic α-Amylase Inhibition

The inhibitory activities of BMG, S.ZnO, Co-ZnO, ZnO/BMG, and Co-ZnO/BMG on murine pancreatic α-amylase enzyme were assessed to determine the suitability of these products to inhibit the activity of metabolically crude active enzymes. Inhibition tests were performed using a synergetic design, considering the impact of cobalt doping and the incorporated BMG substrate. The synthetic Co-ZnO and Co-ZnO/BMG inhibited the activity of α-amylase enzyme (crude active enzyme) by 68.7 ± 1.9% and 84.3 ± 1.8%, respectively (Figure 6B). This demonstrates their higher anti-diabetic activities as compared to acarbose (61.4 ± 1.5%) and miglitol (11.2 ± 1.6%), commonly used commercial drugs and controls (Figure 6B). Additionally, the prepared S.ZnO (38.5 ± 1.87%) and ZnO/BMG structures (54.4 ± 1.9%) exhibited enhanced inhibition effects on the α-amylase enzyme compared to BMG (29.2 ± 1.1%), C.ZnO (9.8 ± 1.1%), and miglitol (18.3 ± 1.4%) (Figure 6B). These experimental findings reflect the significant potential of Co-ZnO and Co-ZnO/BMG as enhanced and effective anti-diabetic agents against the commercial α-amylase enzyme and the metabolically crude active enzyme.

#### 2.3.3. Pancreatic α-Glucosidase Inhibition

**Figure 7:**
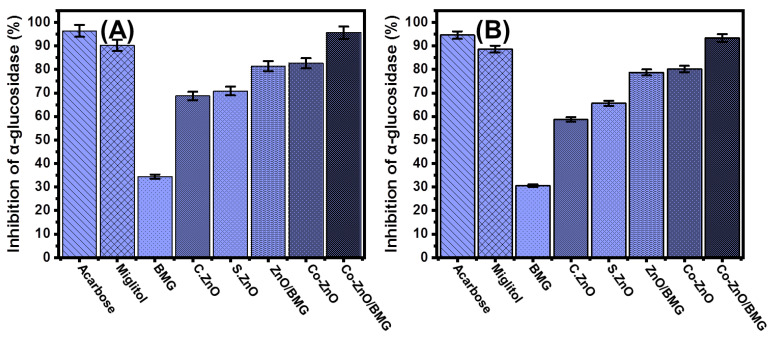
The α-glucosidase inhibition activities of BMG, commercial ZnO (C.ZnO), synthetic ZnO (S.ZnO), Co-ZnO, ZnO/BMG, and Co-ZnO/BMG structures; (**A**) pancreatic α-glucosidase enzyme; and (**B**) murine intestinal α-glucosidase.

The inhibitory effects of BMG, S.ZnO, Co-ZnO, ZnO/BMG, and Co-ZnO/BMG on α-Glucosidase enzyme were followed to control its level as an essential and highly effective enzyme during the metabolic reactions of dietary starch and carbohydrates. Therefore, the development of effective inhibition gents against the α-Glucosidase enzyme can regulate and control the absorption rates of glucose compounds into the blood and significantly decrease hyperglycemia [16]. The inhibition activities of Co-ZnO (82.6 ± 1.6%) and Co-ZnO/BMG (95.7 ± 1.4%) against the pancreatic α-Glucosidase enzyme suggest promising anti-diabetic potential of the Co-ZnO/BMG compared with that of miglitol (90.2 ± 1.3%) and the separate phase of ZnO and BMG investigated in this study (Figure 7A). Moreover, the detected inhibition percentage is close to the determined effect of acarbose (96.4 ± 1.4%) (Figure 7A). The assessed S.ZnO (70.8 ± 1.5%) and ZnO/BMG structures (81.4 ± 1.3%) exhibited enhanced inhibition effects compared to BMG (34.3 ± 1.6%) and C.ZnO (68.7 ± 1.4%) (Figure 7A). These previously reported behaviors agree with the results presented in the literature regarding the synergetic effect of transition metal doping processes and the application of an effective substrate on the bioactivity of ZnO, either as an antioxidant or an anti-diabetic agent.

#### 2.3.4. Murine Intestinal α-Glucosidase Inhibition

The inhibitory effects of BMG, S.ZnO, Co-ZnO, ZnO/BMG, and Co-ZnO/BMG on the active crude intestinal α-Glucosidase enzyme were evaluated in addition to the previously tested commercial forms or more realistic investigations. The recognized results show notable inhibition influences of both Co-ZnO (80.2 ± 1.6%) and Co-ZnO/BMG (93.4 ± 1.8%) on the crude intestinal α-Glucosidase enzyme (Figure 7B). The activity of Co-ZnO/BMG was significantly higher than the determined effects of miglitol (88.6 ± 1.4%) and close to the determined activity of acarbose (94.6 ± 1.34%) drug (Figure 7B). The synthetic S.ZnO (65.6 ± 1.2%) and ZnO/BMG structures (78.8 ± 1.3%) exhibited enhanced inhibition effects as compared to BMG (30.6 ± 1.2%) and C.ZnO (58.7 ± 1.4%) (Figure 7B). The previously determined activities of synthetic ZnO and its composite with BMG (ZnO/BMG) were adequate based on the reported adverse side effects of common drugs as well as their high production costs. Generally, ZnO nanoparticles exhibit promising anti-diabetic activity and contribute to considerably reducing blood glucose levels. Moreover, synthetic ZnO nanoparticles demonstrated a remarkable enhancement of insulin receptors, glucokinase activity, glucokinase genes, and serum insulin [58].

#### 2.3.5. Amyloglucosidase Inhibition

**Figure 8:**
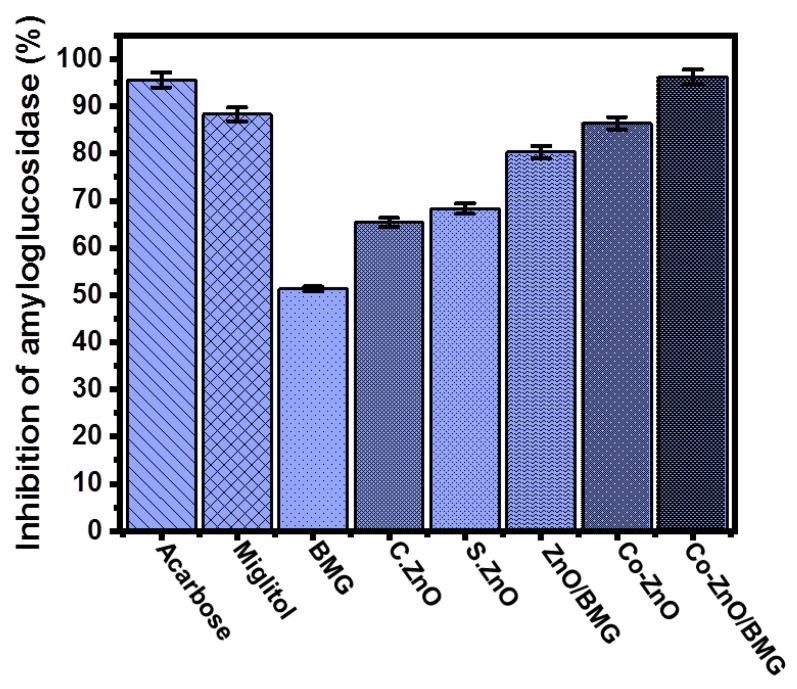
The amyloglucosidase inhibition activities of BMG, commercial ZnO (C.ZnO), synthetic ZnO (S.ZnO), Co-ZnO, ZnO/BMG, and Co-ZnO/BMG structures.

The amyloglucosidase enzyme is also very effective during the breakdown of complex carbohydrates as well as the absorption of the obtained simple compounds. Therefore, inhibition of amyloglucosidases by safe, effective, and low-cost synthetic compounds significantly reduces the transformation performance of complex sugar compounds [59]. The measured amyloglucosidase inhibition percentage using BMG, C.ZnO, S.ZnO, ZnO/BMG, Co-ZnO, and Co-ZnO/BMG was 51.3 ± 1.3%, 65.4 ± 1.3%, 68.3 ± 1.8%, 80.3 ± 1.2%, 86.4 ± 1.2%, and 96.2 ± 1.4%, respectively (Figure 8). However, all the investigated structures (BMG, S.ZnO, Co-ZnO, ZnO/BMG, and Co-ZnO/BMG) exhibited considerable inhibition effects on the amyloglucosidase enzyme, but only the Co-ZnO/BMG multifunctional composite had higher activity than that of miglitol (88.3 ± 1.8%) and acarbose (95.6 ± 1.7%) (Figure 8). Therefore, a multifunctional composite that involves doping ZnO with cobalt over a BMG substrate (Co-ZnO/BMG) is recommended as an enhanced anti-diabetic agent that is characterized by low production cost, high biocompatibility, few side effects, and strong inhibition of common oxidative enzymes.

## 3. Materials and Methods

### 3.1. Materials and Chemicals

The chemical precursors used during the fabrication were cobalt nitrate hexahydrate (Co(NO_3_)_2_·6H_2_O) (98% purity; Sigma-Aldrich, Egypt), zinc nitrate hexahydrate (Zn(NO_3_)_2_·6H_2_O) (98% purity; Sigma-Aldrich, Egypt), and an ammonia solution (33% purity; Sigma-Aldrich, Egypt). Brown macroalgae (*Sargassum* species), which were used as the substrate for the evaluated metal oxides and incorporated into the biological assays, were collected from Ras Ghareb, Red Sea, Egypt. l-ascorbic acid, α-amylase, 2,2′-azino-bis(3-ethylbenzothiazoline-6-sulphonic) acid (ABTS), starch, α-Glucosidase, 1,1-diphenyl-2-picrylhydrazyl (DPPH), para-nitrophenyl α-glucopyranoside (pNPG), and saline phosphate buffer were the essential biological and chemical reagents applied during the antioxidant and anti-diabetic tests. All these reagents were purchased from Sigma-Aldrich, Egypt.

### 3.2. Synthesis of the Tested Structures

#### 3.2.1. Preparation of Incorporated Brown Macroalgae (BMG)

The collected brown macroalgal (*Sargassum*) samples were washed thoroughly with distilled water for four cycles of approximately 15 min to ensure effective removal of the attached salts and associated debris. The washed biomass products were then dried gently in an oven for approximately 24 h at 50 °C to ensure complete dehydration. Afterward, the dehydrated products were ground into smaller pieces using a domestic handheld blender and then transferred into a ball mill (Planetary Ball Mill PM 400) to obtain microfractions of the algae. Then, the microfractions were sieved, and the obtained fractions within the range from 50 to 200 µm (95% < 180, 75% < 120, 50% < 100, and 25% < 60 µm) were used in the further experimental procedure.

#### 3.2.2. Synthesis of ZnO and Co-ZnO Composites with BMG (ZnO/BMG and Co-ZnO/BMG)

**Figure 9:**
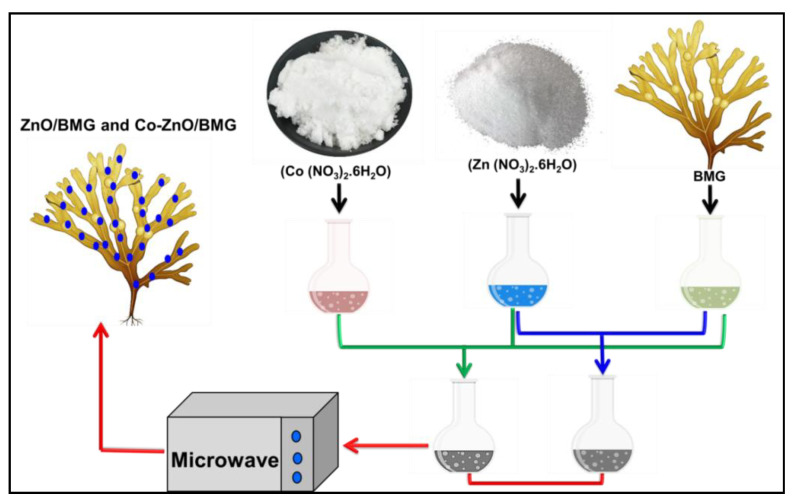
Schematic diagram for the synthesis of ZnO/BMG and Co-ZnO/BMG composites.

The synthesis of ZnO and Co-doped ZnO, both as separated phases or as composites with BMG, was performed using the microwave hydrothermal-assessed precipitation method. Approximately 0.153 g of the main Zn precursor (zinc nitrate salt) was dissolved in 40 mL of distilled water in a Teflon autoclave as a separate synthesis mixture, while another synthesis system was prepared by co-dissolving the zinc-bearing salt (Zn(NO_3_)_2_·6H_2_O), and a cobalt-bearing salt (Co(NO_3_)_2_·6H_2_O) in a Teflon-lined autoclave. The dissolved quantities were adjusted to obtain 4 wt % of both ZnO and Co-ZnO, and the doped parent cobalt was 1 wt %. After the homogenization period reached 60 min, the systems were treated with ammonium hydroxide solution (1 M), which was added gently to separate the synthesis mixtures with regular detection of the solution pH until reaching a pH of 10.5. Subsequently, the two mixtures were stirred for an additional 120 min before being transferred to round-bottom flasks. The flasks were subjected to domestic microwave irradiation at an adjusted power of 700 W for 15 min. Then, the synthesized ZnO and Co-ZnO solid particles were extracted, washed using distilled water to get rid of the residual ammonium ions, and dried overnight at 60 °C. The synthesis of the ZnO/BMG and Co-ZnO/BMG composites involved the incorporation of algae (1 g) during the starting step of the production process. The ground fractions of the algae were dispersed and homogenized within the precursor solutions of zinc nitrate, and the complex zinc nitrate/cobalt nitrate in two individual synthesis mixtures, and they were mixed using a magnetic stirrer for 120 min. Subsequently, the two mixtures were subjected to the same synthesis procedures as those used for pure ZnO and Co-ZnO, including the synthesis pH (10.5), microwave irradiation (700 W; 15 min), washing, and drying stages (12 h; 60 °C). Finally, all products were kept in specific containers, labeled, and used in characterization and biological assay tests. The synthesis procedure is illustrated in Figure 9.

### 3.3. Characterization Techniques

The formed crystal phases and their structural properties were assessed based on the X-ray diffraction patterns of these materials obtained using PANalytical XRD diffractometer (Empyrean) with a Cu-Ká radiation source considering the measuring range between 5° and 80°. The chemical composition was assessed based on the EDX elemental composition using the energy-dispersive X-ray (EDX) technique and the X-ray photoelectron spectra, which were determined by ESCALAB MK II spectrometer with Al anode X-ray exciting source (Al Ká = 1487.6 eV). In addition, the essential chemical functional groups were identified from the Fourier-transform infrared (FT-IR) spectra of the studied materials, which were obtained using a Fourier Transform Infrared spectrometer (FTIR−8400S). The surface forms of the synthesized structures and their general morphologies were described based on their SEM images, which were obtained using a scanning electron microscope (Gemini-Zeiss, Ultra 55). The internal features and nature of the integration processes were evaluated based on the HRTEM images of the synthetic materials, which were obtained using a transmission electron microscope (JEOL-JEM, 2100). In addition to the porous properties, the texture of the surface areas was determined using a Beckman Coulter surface area analyzer (SA3100 type) based on the N_2_ adsorption/desorption curves of the materials.

### 3.4. Antioxidant Studies

#### 3.4.1. Scavenging of Nitric Oxide Radical (NOR)

The scavenger potentialities of ZnO, Co-ZnO, ZnO/BMG, and Co-ZnO/BMG for NOR radicals were determined using an assay described by Kitture et al. [60]. All assessed materials were mixed separately with sodium nitroprusside (2 mL; 10 mM) in pre-prepared phosphate buffer solutions with pH 7.4 (500 μL). The obtained mixtures were incubated separately at 25 °C for 150 min. After incubation, the mixtures were mixed with 500 mL of sulphanilic acid (1 M) and then incubated for another short period (5 min). The mixture was then mixed with naphthyl ethylenediamine dihydrochloride (0.1% *w*/*v*, 1 mL) and incubated for 30 min. Finally, the absorbance of the tested mixtures was determined using a microplate reader at 540 nm and compared with that of the control solutions, and the results were used to calculate the scavenging percentage using Equation (1).

(1) $$Scavenging\;\left(\%\right)=\frac{A540_{Control}-A540_{Test}}{A540_{Control}}\times 100$$

#### 3.4.2. Scavenging of the DPPH Radical

The scavenging assay described by Robkhob et al. [6] was used to evaluate the potentialities of ZnO, Co-ZnO, ZnO/BMG, and Co-ZnO/BMG as scavengers for the DPPH radical. The prepared materials (100 μg/mL; 20 μL) were mixed separately with 80 μL of methanolic solutions, which were supplemented with the tested DPPH radicals (100 μM) using specific 96 well plates. Then, the obtained mixtures were incubated separately for 20 min in the dark, and the absorbance of tested mixtures was determined by a microplate reader at 517 nm and compared to that of the control solutions and the results were used to calculate the scavenging percentage using Equation (2).

(2) $$Scavenging\;\left(\%\right)=\frac{A517_{Control}-A517_{Test}}{A517_{\mathrm{Control}}}\times 100$$

#### 3.4.3. Scavenging of ABTS Radical

The scavenging assay described by Dappula et al. [49] was used to evaluate the potentialities of ZnO, Co.ZnO, ZnO/BMG, and Co-ZnO/BMG as scavengers for ABTS radical. The ABTS solutions used during the tests were prepared by simple dissolution of the ABTS compound (44 mg) in deionized water (10 mL). These solutions were supplemented separately with potassium persulfate (3 mM) as an essential step to generate free cations (ABTS^●+^) of the ABTS radical. This reaction was conducted in the dark at 25 °C for up to 18 h. Then, the reaction mixtures were diluted with methanol at a ratio of 1:29 to obtain fresh ABTS^●+^. Subsequently, the synthetic materials were mixed separately (100 μg/mL) with the previously prepared ABTS solutions (290 μL), and the mixtures were kept for 30 min. The absorbance of the tested mixtures was determined using a microplate reader at 734 nm and compared to that of the control solutions, and the results were used to calculate the scavenging percentage using Equation (3).

(3) $$Scavenging\;\left(\%\right)=\frac{A734_{Control}-A734_{Test}}{A734_{Control}}\times 100$$

#### 3.4.4. Scavenging of Superoxide Radical

The scavenging assay described by Robkhob et al. [6] was applied to evaluate the potentialities of ZnO, Co.ZnO, ZnO/BMG, and Co-ZnO/BMG as scavengers for superoxide radical (O^●−^). The prepared materials (100 μL) were mixed separately with previously prepared mixtures of EDTA (200 μL; 12 mM), ethanol (200 μL), riboflavin (100 μL; 20 μg), and NBT (100 μL; 0.1 mg). The products of the previous step were supplemented separately with a phosphate buffer solution (3 mL) and exposed to a light source for approximately 5 min. Subsequently, the absorbance of the tested mixtures was determined using a microplate reader at 540 nm and compared with that of the control solutions, and the results were used to calculate the scavenging percentage using Equation (1).

### 3.5. Anti-Diabetic Studies

#### 3.5.1. Inhibition Assay of Porcine Pancreatic α-Amylase

The inhibition assay described by Robkhob et al. [6] was used to evaluate the potential of ZnO, Co-ZnO, ZnO/BMG, and Co-ZnO/BMG as inhibition agents against the commonly tested commercial pancreatic α-amylase enzyme. The prepared materials were mixed separately at 100 μg/mL with the investigated α-amylase enzyme (50 μg/mL), and the obtained mixtures were subjected to an incubation process for 10 min at 37 °C. Afterward, the mixtures were supplemented with a starch substrate (1%), and the absorbance was determined by a microplate reader at 540 nm and compared with that of the control solutions, and the results were used to calculate the inhibition percentage using Equation (4).

(4) $$Inhibition\;\left(\%\right)=\frac{A540_{Control}-A540_{Test}}{A540_{\mathrm{Control}}}\times 100$$

#### 3.5.2. Inhibition Assay of Crude Murine Pancreatic α-Amylase

This assay was performed to ensure the suitability of ZnO, Co-ZnO, ZnO/BMG, and Co-ZnO/BMG to inhibit crude active enzymes in comparison with commercially available enzymes. Crude enzymes were extracted from the pancreas of a 10-week-old Swiss male mouse. The mouse was subjected to starvation for approximately 12 h. Then, the starved pancreas was excised and immersed carefully in a phosphate buffer solution containing protease inhibitors. Next, the cell-free supernatant was extracted by a fast centrifugation process (15 min; 10,000 rpm), and the mixture was diluted regularly until the detection of 0.4 by the microplate reader at a wavelength of 280 nm. Under these conditions, the pancreas can be used as a source of the active crude enzyme, and the inhibition assay of the synthetic materials was performed following the procedures described in Section 3.5.1.

#### 3.5.3. Inhibition Assay of α-Glucosidase

The inhibition assay described Sanap et al. [61] was used to evaluate the potentialities of ZnO, Co.ZnO, ZnO/BMG, and Co-ZnO/BMG as inhibition agents against the commercial form of the α-Glucosidase enzyme. The prepared materials were mixed separately at 100 μg/mL with the investigated α-Glucosidase enzyme (100 μL; 0.1 unit/mL), and the resulting mixtures were incubated for 60 min at 37 °C. Subsequently, the mixtures were supplemented with pNPG (10 mL) before re-incubation for an additional 10 min. Then, the Na_2_CO_3_ solution (0.1 M; 2 mL) was added to the mixtures to stop the reactions. Finally, the absorbance of the nitrophenol released from pNPG within the investigated mixtures was determined using a microplate reader at 420 nm and compared with that of the control solutions, and the results were used to calculate the inhibition percentage using Equation (5).

(5) $$Inhibition\;\left(\%\right)=\frac{A420_{Control}-A420_{Test}}{A420_{Control}}\times 100$$

#### 3.5.4. Inhibition Assay of Crude Murine Intestinal α-Glucosidase

The crude intestinal α-Glucosidase enzyme was isolated according to the previously reported procedure during the extraction of the crude α-amylase enzyme in Section 3.5.2. The inhibition assays of ZnO, Co.ZnO, ZnO/BMG, and Co-ZnO/BMG against the crude intestinal α-Glucosidase enzyme were performed following the same procedures described in Section 3.5.3 incorporating p-nitrophenyl-α-D-glucopyranoside as substrate.

#### 3.5.5. Amyloglucosidase Inhibition Assay

The inhibition assay described by Lawande et al. [62] was used to evaluate the potential of ZnO, Co-ZnO, ZnO/BMG, and Co-ZnO/BMG as inhibitors of the amyloglucosidase enzyme. The prepared materials were mixed separately at 100 μg g/mL with the investigated amyloglucosidase enzyme (0.1 unit/mL), and the resulting mixtures were incubated for 10 min at 37 °C with 1% of starch substrate. Subsequently, the absorbance of the mixtures was determined using a microplate reader at 540 nm and compared with that of the control solutions, and the results were used to calculate the scavenging percentage using Equation (4).

## 4. Conclusions

ZnO/brown macroalgae (ZnO/BMG) and cobalt-doped ZnO/brown macroalgae (ZnO/BMG) composites were synthesized and characterized as potential antioxidant and anti-diabetic agents. The Co-ZnO/BMG composite showed the best biological activity, both as an antioxidant and anti-diabetic agent. It exhibited excellent scavenging properties for the common free reactive oxygen radicals (DPPH (89.6 ± 1.5%), nitric oxide (90.2 ± 1.3%), ABTS (87.7 ± 1.8%), and O_2_^●−^ (46.7 ± 1.9%)). Moreover, it showed strong inhibition effects on the essential oxidative enzymes (porcine α-amylase (90.6 ± 1.5%), crude α-amylase (84.3 ± 1.8%), pancreatic α-Glucosidase (95.7 ± 1.4%), crude intestinal α-Glucosidase (93.4 ± 1.8%), and amyloglucosidase (96.2 ± 1.4%). These results suggests that the Co-ZnO/BMG multifunctional composite can be used as an enhanced antioxidant and anti-diabetic agent, considering the determined efficiencies of common drugs (miglitol and acarbose) and their expected side effects.

## Data Availability

Data are available upon reasonable request, by the Corresponding Authors.

## Recommendation

Further studies will be conducted involving the in-vivo investigation of the antioxidant and anti-diabetic activities of ZnO/BMG and Co-ZnO/BMG based on the different physiological effects as well as the safety and toxicity of the synthetic structures.
